# Severe progressive scoliosis due to huge subcutaneous cavernous hemangioma: A case report

**DOI:** 10.1186/1748-7161-6-3

**Published:** 2011-03-17

**Authors:** Yoji Ogura, Kota Watanabe, Naobumi Hosogane, Takashi Tsuji, Ken Ishii, Masaya Nakamura, Yoshiaki Toyama, Kazuhiro Chiba, Morio Matsumoto

**Affiliations:** 1 Department of Orthopaedic Surgery, Keio University School of Medicine, Tokyo, Japan; 2 Department of Advanced Treatment for Spine and Spinal Cord Disorders, Keio University School of Medicine, Tokyo, Japan

## Abstract

Cavernous hemangioma consists mainly of congenital vascular malformations present before birth and gradually increasing in size with skeletal growth. A small number of patients with cavernous hemangioma develop scoliosis, and surgical treatment for the scoliosis in such cases has not been reported to date. Here we report a 12-year-old male patient with severe progressive scoliosis due to a huge subcutaneous cavernous hemangioma, who underwent posterior correction and fusion surgery. Upon referral to our department, radiographs revealed a scoliosis of 85° at T6-L1 and a kyphosis of 58° at T4-T10. CT and MR images revealed a huge hemangioma extending from the subcutaneous region to the paraspinal muscles and the retroperitoneal space and invading the spinal canal. Posterior correction and fusion surgery using pedicle screws between T2 and L3 were performed. Massive hemorrhage from the hemangioma occurred during the surgery, with intraoperative blood loss reaching 2800 ml. The scoliosis was corrected to 59°, and the kyphosis to 45° after surgery. Seven hours after surgery, the patient suffered from hypovolemic shock and disseminated intravascular coagulation due to postoperative hemorrhage from the hemangioma. The patient developed sensory and conduction aphasia caused by cerebral hypoxia during the shock on the day of the surgery. At present, two years after the surgery, although the patient has completely recovered from the aphasia. This case illustrates that, in correction surgery for scoliosis due to huge subcutaneous cavernous hemangioma, intraoperative and postoperative intensive care for hemodynamics should be performed, since massive hemorrhage can occur during the postoperative period as well as the intraoperative period.

## Background

Cavernous hemangioma consists mainly of congenital vascular malformations, which is present before birth and gradually increasing in size with skeletal growth. The vertebral body is one of the most commonly recognized sites of cavernous hemangioma, and found incidentally on radiological imaging[1,2]. While many cases of hemangioma are asymptomatic, others present with pain or spinal deformity, and those located within the spinal canal may present with neurological disorders. There are several reports of scoliosis secondary to cavernous hemangioma originating in the vertebral body [3-6], and one of scoliosis secondary to a subcutaneous cavernous hemangioma[7]. However, to our knowledge, there are no reports of surgical treatment for these conditions. Here we report a case of scoliosis caused by a huge subcutaneous cavernous hemangioma that was surgically treated.

## Case presentation

**Figure 1:**
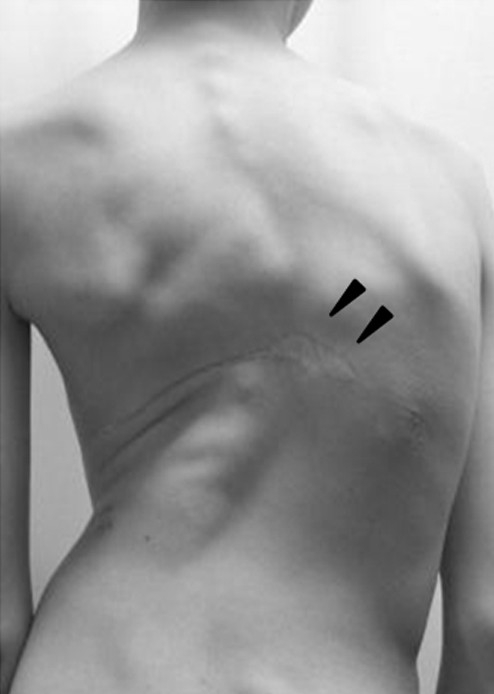
**Clinical appearance of the patient**. On physical examination, significant protrusion of the right ribs, as well as a huge subcutaneous tumor measuring approximately 15 cm in diameter and a surgical wound measuring approximately 5 cm in length on the left back (arrowheads) were recognized.

A 12-year-old boy with progressive trunk deformity and a huge subcutaneous tumor was referred to our department. At the age of three, the subcutaneous tumor was found in the back, and biopsy of the tumor indicated a diagnosis of cavernous hemangioma. During that biopsy, a massive hemorrhage occurred, and the patient required a blood transfusion (3000 ml). Scoliosis was diagnosed at age 11, and the patient underwent brace treatment. However, the scoliosis worsened progressively, and the patient was finally referred to our department. Upon physical examination, we found significant protrusion of the right back ribs. On the left back was a huge subcutaneous tumor, measuring approximately 15 cm in diameter, and a scar from a previous surgical wound that was approximately 5 cm long (Figure 1). The mass was not tender or throbbing, and no neurological abnormalities were observed.

**Figure 2:**
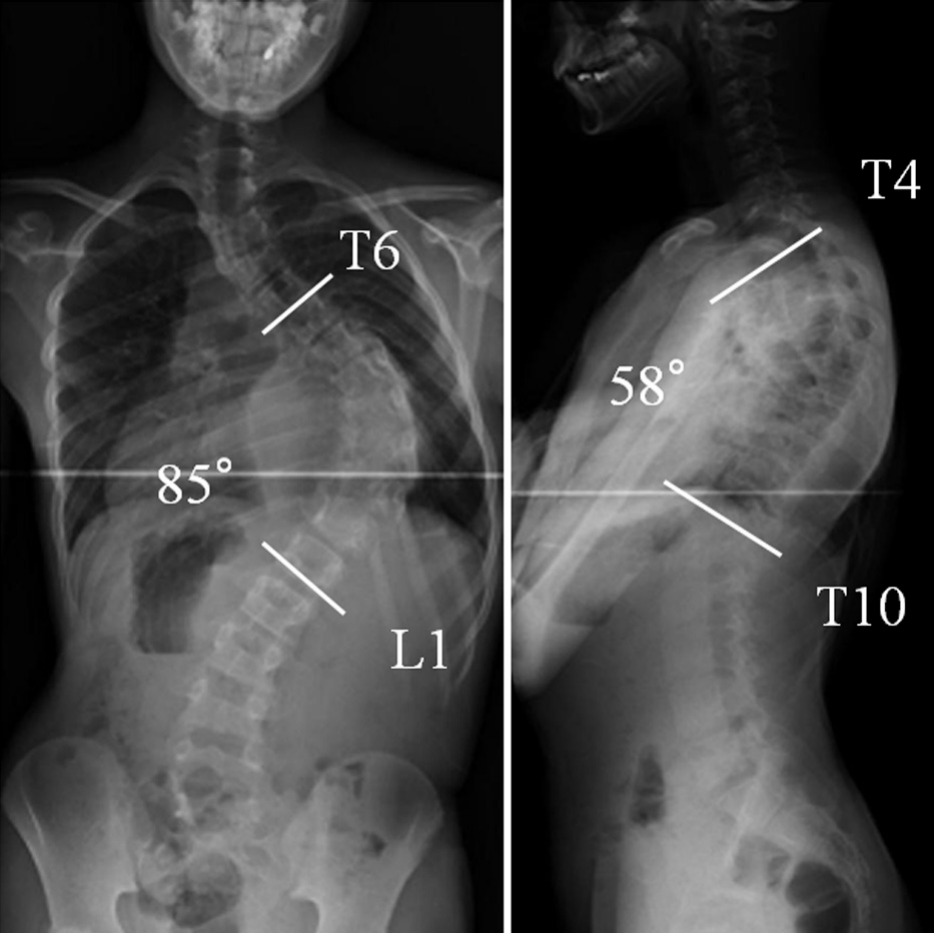
**Standing X-ray films**. Radiographs indicated a scoliosis of 85° at T6-L1 and a kyphosis of 58° at T4-T10. The Risser sign was grade zero, and the triradiate cartilages were open.

**Figure 3:**
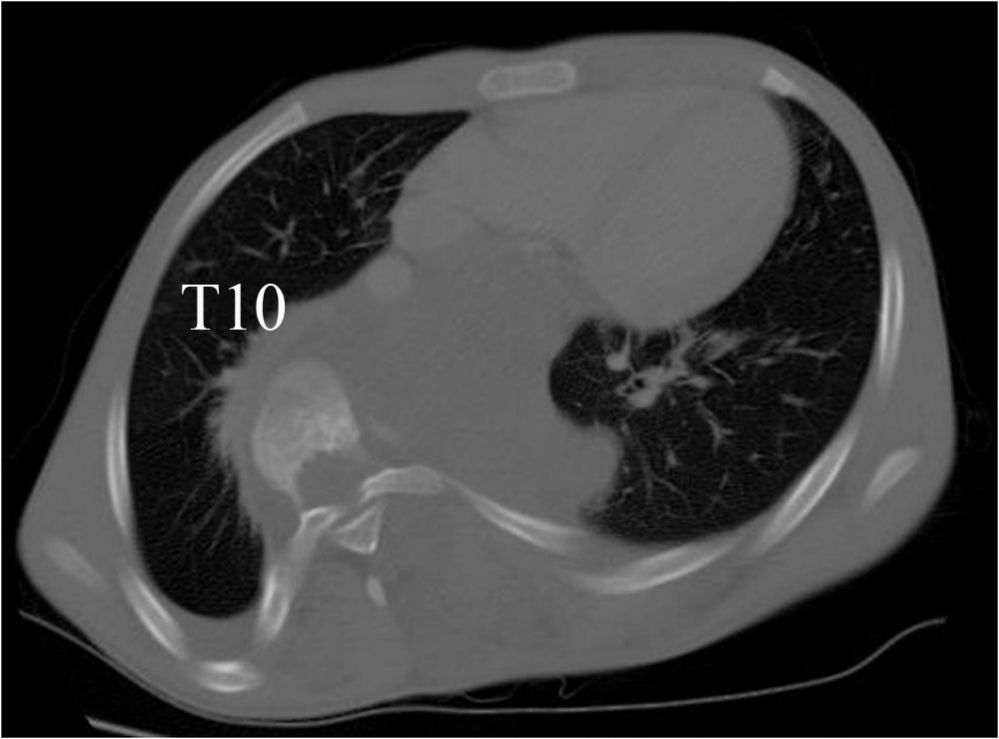
**Axial CT image**. Axial CT image at T10 indicating the atrophy of vertebral bodies and invasion of the hemangioma.

**Figure 4:**
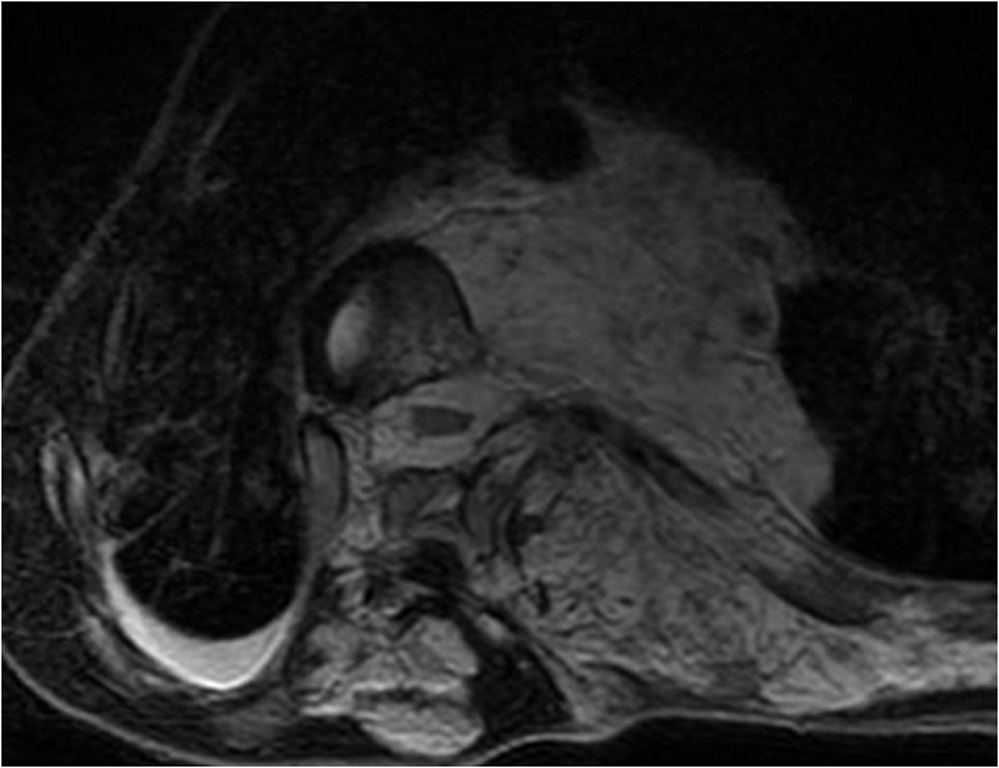
**MR images of the hemangioma**. The huge hemangioma extending from the subcutaneous region to the paraspinal muscles and the retroperitoneal space was observed at T6-L1 primarily on the left side. Invasion into the spinal canal and compression of the dura by the hemangioma were also recognized.

Full-length, standing radiographs demonstrated a scoliosis of 85° at T6-L1 and a kyphosis of 58° at T4-T10. The Risser sign was grade zero, and the triradiate cartilages were open (Figure 2). Traction radiography showed a correction rate of 6%, indicating extremely low flexibility. On the axial CT images, vertebral body atrophy was recognized at T7-T10, where the vertebral bodies were surrounded by the cavernous hemangioma (Figure 3). No congenital deformity of the vertebral bodies was observed. MR images revealed that the hemangioma extended from the subcutaneous region to the paraspinal muscles and the retroperitoneal space at T6-L1, primarily on the left side. Invasion of the hemangioma into the spinal canal and compression of the dura by the hemangioma were recognized at the T8-T10 level (Figure 4).

The diagnosis of severe progressive scoliosis associated with cavernous hemangioma was made. Prior to the surgery, we decided against the use of radiotherapy, which may reduce the size of the tumor or blood circulation through it, because irradiation of the spine can lead to pseudoarthrosis. In planning the surgery, an anterior approach was considered first, since the hemangioma was mainly located on the concave side of the curve. However, we abandoned this idea because damage to the hemangioma during disc removal or screw placement would have required us to manage the hemorrhage from inside the narrow thoracic cage, which might not have been successful. Although bleeding from hemangioma was inevitable if we used the posterior approach, we had the option of stopping the bleeding by the tamponade effect of closing the wound. Therefore, we used the posterior approach for correction surgery and fusion with pedicle screws at T2-L3.

**Figure 5:**
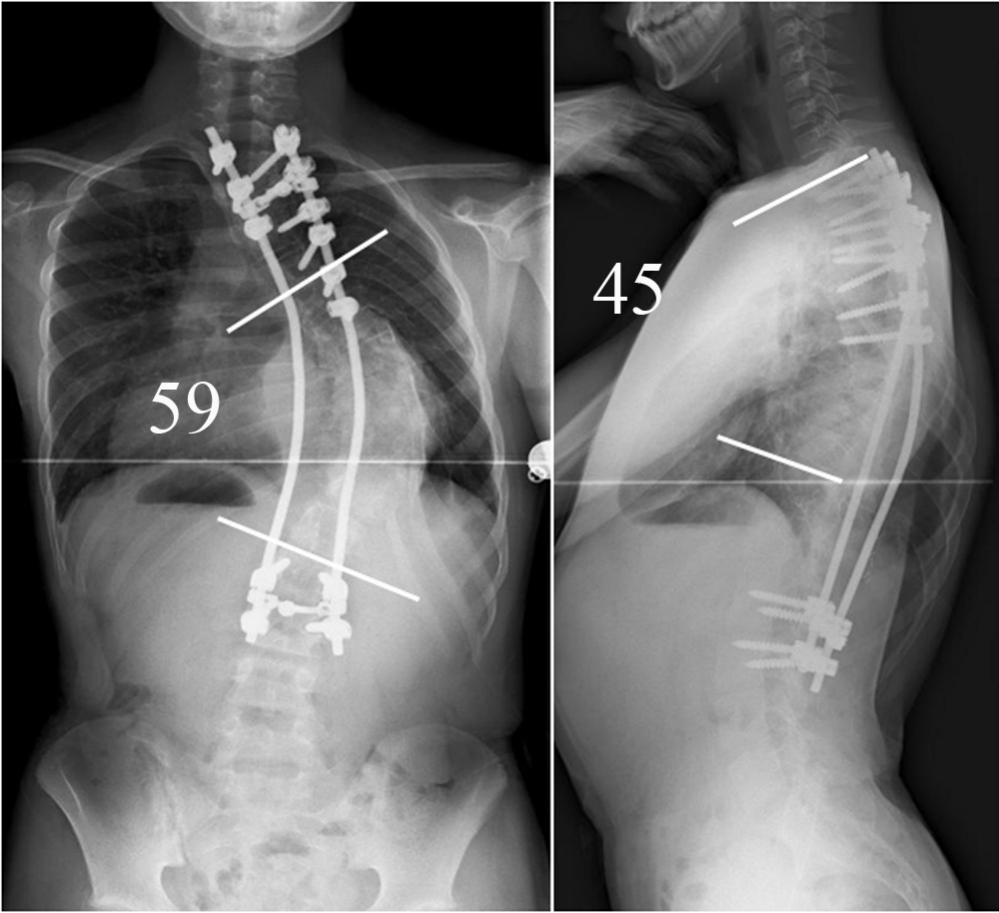
**Postoperative radiographs**. After surgery, scoliosis was corrected to 59°, and kyphosis to 45°, with correction rates of 31% and 22%, respectively.

A midline skin incision was made on the patient's back. Soon after the incision was made, the hemangioma hemorrhaged massively, and hemostasis by coagulation failed using bipolar forceps, electrocautery, and ligation. Thus, the surgery was continued with gauze packing at the bleeding sites. The soft tissues were carefully detached from the spinous processes or laminae subperiosteally using a spatula to minimize damage to the pseudocapsule of the hemangioma, and consequently, the hemorrhage greatly decreased. Pedicle screws were placed segmentaly, except from T8 to T10, where the hemangioma invaded the spinal canal, to avoid spinal cord injury from intracanalar hemorrhage of the hemangioma. Since we encountered massive hemorrhage at T11, 12 and L1, we could not place pedicle screws at these levels, where we had hoped to place them to increase the number of anchor points. The correction force was applied carefully to avoid screw failure, since fewer screws could be placed than were needed and the flexibility of the curves was quite low. After the correction, abundant bone grafting was performed using the iliac crest and local bone. No drainage tube was placed inside the wound, since it could have increased the risk of postoperative hemorrhage by penetrating or damaging the surrounding hemangioma (figure 4) during insertion of the tube. After surgery, the scoliosis was corrected to 59°, and kyphosis to 45°, with correction rates of 31% and 22%, respectively (Figure 5). The intraoperative time was 314 minutes, and the intraoperative blood loss was 2800 ml.

**Figure 6:**
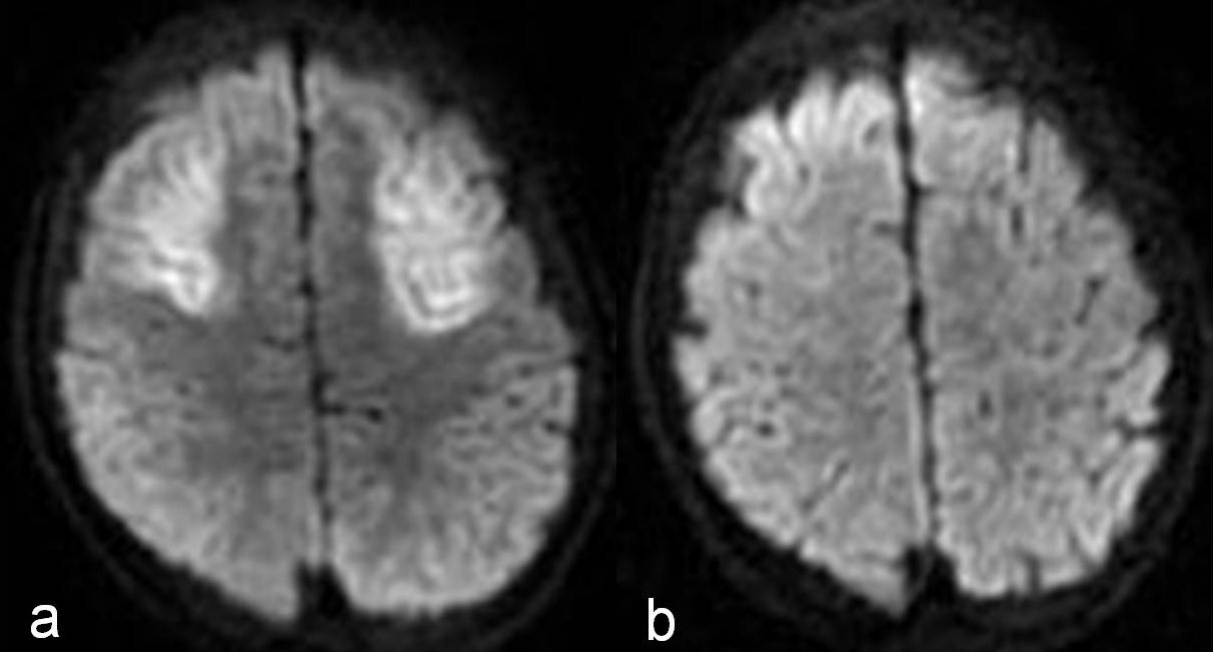
**MRI indicating brain ischemia**. a: MRI revealed ischemic lesions in the bilateral frontal and temporal lobes. These lesions caused postoperative sensory and conduction aphasia. b: MRI revealed that the ischemic lesions had become much smaller.

The patient's hemodynamics stabilized with blood hemoglobin level of 9.2 mg/dl when the surgery finished at 7 pm after the transfusion of 400 ml of preoperative donated autologous blood and 1350 ml of intraoperative and postoperative cell saver autologous blood. The patient was admitted to intensive care unit at 8 pm. At 11 pm (four hours after surgery), his blood pressure was 92/52 mmHg with stable hemodynamics, and his blood hemoglobin level was 8.5 mg/dl. However, seven hours after surgery, after the administration of diazepam to control agitation, his blood pressure suddenly decreased to an unmeasurable level, and the patient became unconscious with apnea. At that time, the hemoglobin level was 5.9 mg/dl. Endotracheal intubation was immediately performed, along with blood transfusion, which resulted in his recovery from hypovolemic shock. The following day, however, the patient developed disseminated intravascular coagulation (fibrinogen degradation products [FDP] 32.3 μg/ml, platelet count 95,000/μl, prothrombin time international normalized ratio [PT-INR] 2.28), and received fresh frozen plasma and gabexate mesylate. Seven days after the surgery, when his general condition had stabilized, the patient was extubated. However, as the patient became conscious, a language disorder became apparent, and a head MRI revealed ischemic lesions in the bilateral frontal and temporal lobes. Sensory and conduction aphasia was diagnosed, caused by cerebral hypoxia during the hypovolemic shock on the day of the surgery (Figure 6a). The patient gradually recovered from the aphasia, and the ischemic lesions became smaller on MRI (Figure 6b). At present, two years after the surgery, although the patient has completely recovered from the aphasia.

## Discussion

To our knowledge, there has been only one report of scoliosis caused by an enlarging subcutaneous cavernous hemangioma, which was reported by Lozman et al. The case was that of a 13-year-old boy with a scoliosis of 55°. Although surgical removal of the hemangioma was attempted, the procedure was halted after only a biopsy was completed, due to massive hemorrhage. Treatment using a Milwaukee brace was then undertaken, and fortunately, the patient showed no progression of the scoliosis for 15 months of follow-up [7]. Because cavernous hemangiomas invading the spinal epidural space are often seen in Klippel-Trenaunay syndrome, we initially suspected an association with this disease. However, since port wine stain (cutaneous hemangiomas), varicosities, and hypertrophy of the affected limbs are characteristics of Klippel-Trenaunay syndrome, but were not seen in the present case, we have reported this case as solely a cavernous hemangioma. In general, asymptomatic cavernous hemangioma is not an indication for surgery, since uncontrollable hemorrhage can occur during the operation [8]. Some reports have described treating hemangiomas with vascular embolization or radiotherapy, concluding that enlargement or relapse of the hemangiomas could be controlled without surgery [3,5-7,9]. Asumu et al. reported a patient with scoliosis caused by cavernous hemangioma originating in the vertebral body. The Cobb angle of the curve was 35°, and the patient was treated by vascular embolization, without exacerbation of the neurologic symptoms or an increase in the Cobb angle, yielding a favorable result. The authors concluded that vascular embolization may be a useful option when a single vasa vasorum is identifiable [3]. Bucknill also reported a favorable result with ligation of the vasa vasorum followed by radiotherapy combined with posterior fusion [4].

In our present case of a 12-year-old boy with scoliosis of 85 degrees, posterior correction and fusion surgery were considered necessary, because significant progression of the deformity was recognized despite thorough brace treatment. Preoperative vascular embolization, which is reported to be effective, was not performed for the following reasons. Preoperative contrast-enhanced CT showed only a slight enhancement in part of the tumor, and no enlarged feeding arteries were visible. These are rare findings for hemangioma. We speculated that the blood flow inside the sinusoid was so slow that contrast-enhanced CT could not provide a clear image of the tumor, and expected that we would not be successful in identifying feeding arteries by angiography either. Although preoperative radiotherapy could have reduced the vascularity of the hemangioma, it was not performed because of the increased risk of pseudarthrosis following irradiation. Local radiotherapy also might have reduced the size or blood circulation of the tumor near the operated area. However, to reduce bleeding during correction and fusion surgery of the spinal column, even local radiotherapy would have had to be directed close to the spine, increasing the risk of pseudoarthrosis. Therefore, we decided against its use.

During the surgery, the posterior correction and fusion were performed without removing the hemangioma, since massive hemorrhage associated with removal of the tumor was predicted. As was expected preoperatively, massive hemorrhage from the hemangioma occurred following a breach of the tumor pseudocapsule, and hemostasis was difficult to achieve. However, the intraoperative hemorrhage was reduced to some extent by the cautious subperiosteal detachment of the soft tissues from the posterior bony elements, which prevented damage to the hemangioma. At the time of wound closure, the absence of massive hemorrhage was confirmed. Nevertheless, the patient suddenly entered a state of hypovolemic shock after the administration of diazepam 7 hours postoperatively. We suspect that the reduced circulating blood volume due to the intraoperative massive hemorrhage depleted the blood inside the hemangioma, which reduced the hemorrhage from the hemangioma by the end of the surgery. However, the postoperative blood and fluid transfusion increased the circulating blood volume, causing the reperfusion of the hemangioma, which resulted in hemorrhage from hemangioma and unstable hemodynamics. Since the patient's hemodynamics were stable according to the arterial line monitor, we underestimated the hemorrhage inside the wound and did not pay sufficient attention to the hemoglobin concentration. To monitor the postoperative hemorrhage status more accurately, we should have checked the hemoglobin concentration and coagulation factor levels more frequently, even though the hemodynamics were stable. The disseminated intravascular coagulation that developed one day after surgery was thought to be caused, at least in part, by the large amount of hemorrhage and consumption of coagulation factors inside the huge hemangioma. Transfusion of whole blood and fresh frozen plasma should have been performed earlier to prevent hypovolemic shock. The reasons for the delayed blood transfusion were, first, we overestimated the hemoglobin level and underestimated the postoperative hemorrhage inside the wound. Moreover, we tried to manage the loss of circulating blood volume with autologous blood collected perioperatively. Although this episode of shock may have caused the cerebral hypoxia, the early resuscitation and youth of the patient may have contributed to his gradual recovery from aphasia.

## Conclusions

In the surgical treatment of progressive scoliosis in patients with huge cavernous hemangioma, preoperative embolization should be considered if dominant feeding arteries are recognized on contrast CT or angiography. The use of radiotherapy, which is reported to be effective in reducing hemangioma size, should be considered carefully, since it may increase the risk of pseudoarthrosis. Because a large hemorrhage can occur during the postoperative period as well as intraoperatively, careful perioperative attention should be paid to such patients' hemodynamics by using arterial and central venous line monitoring. Transfusions of whole-blood and/or fresh frozen plasma at volumes that could prevent postoperative hypovolemic shock and coagulopathy should be considered.

## Consent

Written informed consent was obtained from the parents of the patient for publication of this case report and any accompanying images. A copy of the written consent is available for review from the Editor-in-Chief of this journal

## Competing interests

The authors declare that they have no competing interests.

## Authors' contributions

YO, KW and MM made substantial contributions to the conception and design, and the acquisition, analysis, and interpretation of data. They were also involved in drafting and revising the manuscript. NH, TT, KI, and MN contributed to the conception and design, and performed critical revision of the manuscript. YT and KC, performed critical revision of the manuscript and gave final approval of the version to be published.
